# Achieving Critical View of Safety via a New Technique: The Triple One (111) Technique

**DOI:** 10.7759/cureus.44098

**Published:** 2023-08-25

**Authors:** Muhammad Iftikhar, Muhammad Shah, Zia Ullah, Haythem Abdul Shakoor, Shahid Ullah

**Affiliations:** 1 General Surgery Department, Hayatabad Medical Complex Peshawar, Peshawar, PAK; 2 Anatomy Department, Peshawar Institute of Medical Sciences, Peshawar, PAK

**Keywords:** misidentification, laparoscopic cholecystectomy, laparoscopic surgery, post cholecystectomy bile duct injury, complications, vasculobiliary, cvs, lap chole, triple one technique

## Abstract

Background: Misidentification of anatomical structures is one of the most common causes of bile duct injury following laparoscopic cholecystectomy. Achieving Critical View of Safety (CVS) is a standard step in conducting safe cholecystectomy all over the world. In our institute, we achieve CVS via a unique technique called Triple One or 111 and find it very helpful and easy to achieve CVS. Moreover, the rate of conversion has also decreased while achieving CVS via this technique. The unique aspect of the Triple One technique is that by following this method, even new laparoscopic surgeons can achieve CVS very easily in difficult cases and, hence, it decreases the chances of vasculobiliary injury (VBI).

Objective: This study aimed to determine how effective the Triple One technique is in achieving CVS as well as in lessening the chances of VBI.

Material and methods: A total of 545 patients were admitted through the outpatient department, ranging in age from 30 to 70 years, with a mean of 50 years. The study comprised patients with American Society of Anaesthesiologists (ASA) I & II, acute and chronic cholecystitis, and symptomatic cholelithiasis. The study excluded patients with co-morbidities, prior abdominal procedures, and suspected complications. On the second postoperative day, all patients received their discharge papers and on the seventh postoperative day, follow-up was completed.

Results: Successful gallbladder extraction using the Triple One technique was achieved in 540 (99%) cases. The other five (1%) cases converted to open cholecystectomy because of the difficult gallbladder anatomy and extensive scarring. No VBI or bile duct injury was noted. No mortality was recorded during the study period.

Conclusion: By incorporating CVS using the Triple One technique into our policies and curriculum, we may encourage safe cholecystectomy practices and prevent bile duct injuries.

## Introduction

Critical view of safety (CVS) emerges as the universally acclaimed and meticulously standardized technique, revered as the gold standard for upholding patient safety in laparoscopic cholecystectomy (lap chole) all over the world. When performing a laparoscopic cholecystectomy (lap chole), safety is essential, but it can be challenging to ensure it. To ensure that this technique is carried out safely and with the best results, a CVS is necessary [1].

CVS is an anatomical identification technique that targets the cystic duct and the cystic artery [2]. There has been an increase in the occurrence of vasculobiliary injuries (VBI) immediately following the introduction of lap chole, which has led to the acceptance of CVS [3,4]. It is not easy to achieve CVS when the gallbladder has rough scarring around its neck and skeletonization of the cystic structure requires dissection of the proximal one-third of the cystic plate [5]. I

CVS should begin with imaging Rouviere's sulcus and segment IV of the liver, as recommended by the Tokyo Guidelines 2018 for lap chole [6]. The lap chole procedure requires anatomically specific landmarks to be applied to a gallbladder that is difficult to remove. Rouviere's sulcus is also generally considered a landmark associated with the inflow of hepatic fluid into the right hepatic lobe. Rouviere's sulcus is recognizable in only 75% of patients with acute cholecystitis, since omental fusion or inflammation may obscure its visibility. Sometimes it is difficult to identify Rouviére's sulcus due to gallstones impacting the gallbladder's neck in difficult lap chole [7].

We advocate the Triple One (111) method of dissection in the present study. This is a new technique that has never been used previously. The rationale of this study is to develop a method of standard dissection technique in order to achieve CVS and to prevent major VBI that result from the misidentification of cystic structures during lp chole; this will help even new laparoscopic surgeons to achieve perfection in lap chole.

## Materials and methods

This prospective experimental study was conducted at the General Surgery Department of Hayatabad Medical Complex Peshawar, Pakistan. Permission was granted by the Hospital Research and Ethical Review Board of Hayatabad Medical Complex Peshawar (approval number: 11501-3). The Triple One method was used to achieve CVS in all the lap choles.

A total of 545 patients were admitted through the outpatient department, ranging in age from 30 to 70 years, with a mean of ±50 years. The study comprised patients with American Society of Anesthesiologists (ASA) class I & II, acute and chronic cholecystitis, and symptomatic cholelithiasis. The study excluded patients with co-morbidities, prior abdominal procedures, and suspected complications. On the second postoperative day, all patients were discharged and on the seventh postoperative day, follow-up was completed.

Surgical technique

Triple One technique is a name given to a method of dissection used to achieve CVS, which is a standardized recognized step in lap chole all over the world. All surgeons achieve CVS via their own dissecting techniques. We, in our institute, are also achieving CVS via our own technique named Triple One, and we believe that this is one of the safest ways of achieving CVS and it has been developed after a long lap chole experience.

The conventional four-port method was used for all lap chole. In the epigastric region, the operator's 10 mm working port (for the right hand) was inserted. Located along the midclavicular line on the right side of the right subcostal area, a 5 mm port was inserted for the operator's left hand. Through the 10 mm port at the umbilicus, a flexible videoscope measuring 5 mm or 10 mm was inserted. A 5 mm port was placed along the anterior axillary line in the subcostal region for gallbladder retraction. The gallbladder fundus was cranially retracted under the pneumoperitoneum to provide visualization of the hepatic hilar region.

**Figure 1:**
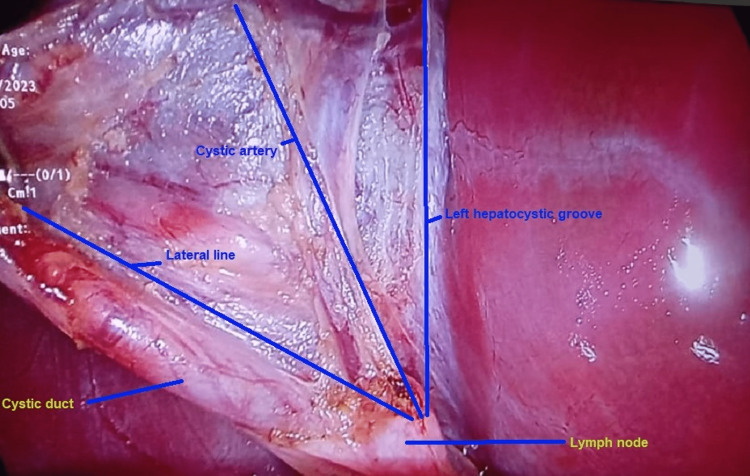
Left hepatocystic groove

**Figure 2:**
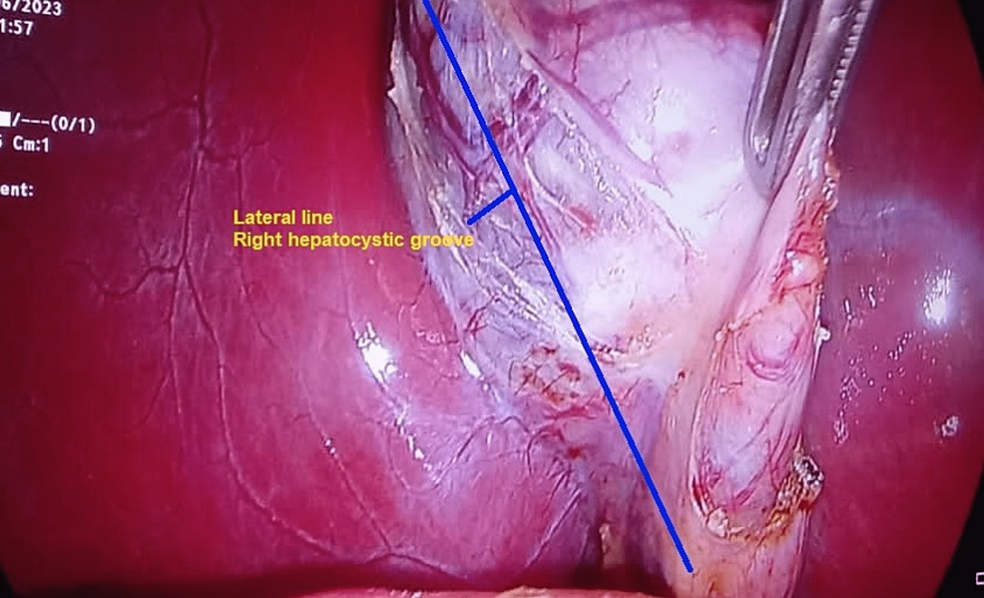
Right hepatocystic groove

In the Triple One method, after dissection, three linear 5 cm incisions are made in the peritoneum covering the gallbladder. Two of the incisions are made on either side of the gallbladder in the hepato-cystic groove while the third incision is made between the cystic duct and artery just lateral to the lymph node of Lund, which is a very commonly visible structure in almost all cases and is usually enlarged in inflamed and difficult cases. The hook cautery is used for these incisions. The cystic duct and cystic artery are always dissected apart. Then the lower ends of these incisions are connected with each other to make a “W”. In this way, the infundibular and cystic duct separates from each other and the gallbladder neck is easy to be elevated from the rest of the biliary structures and duodenum (Figures 1, 2). The steps are given in brief in Table 1.

**Table 1:** Steps of the Triple One (111) technique

Step Number	Step Description
1.	Identify the anatomy and R4U line (Rouviere's sulcus→Segment 4→Umbilical fissure) and stay above it.
2.	Identify the lymph node of Lund. (Lymph node is always present and it is enlarged in inflammation. The main argument of our study is while the anatomy is more difficult in inflammation, the lymph node is much easier to identify in inflammation).
3.	Make three incisions, 5 cm each, on the peritoneum covering the gallbladder.

The trend of dissection in this technique is toward the body of the gallbladder rather than going downward. In this way, there is minimal chance of VBI. In the end, CVS is achieved with only two structures entering the gallbladder.

## Results

The age of patients ranged from 30 to 70 years, with a mean of ±50 years. There were 235 (43.1%) males and 310 (56.9%) females. All patients underwent lap chole with the Triple One technique for achieving CVS. Operative time ranged from 45 minutes to 120 minutes with a mean of 82.5 minutes. Mean intraoperative blood loss was 15 ml (range, 1‐29 ml). No intraoperative or postoperative complications were seen in patients hospitalized. Postoperative hospital stay was one to two days with a mean of 1.5 days (Table 2).

**Table 2:** Patient demographic and other characteristics VBI: vasculobiliary injury; BDI: bile duct injury

Characteristics	Frequency	Percentage
Gender		
Male	235	43.1%
Female	310	56.9%
VBI/BDI	0	0%
Mortality	0	0%
Other characteristics		
Mean age (years)	35 years	
Mean operative (mins)	82.5 mins	
Mean blood loss (ml)	15 ml	
Mean hospital stay (days)	1.5 days	

Chronic cholecystitis was observed in 397 (72.8%) cases, while acute cholecystitis was seen in 148 (27.2%) cases (Figure 2).

**Figure 3:**
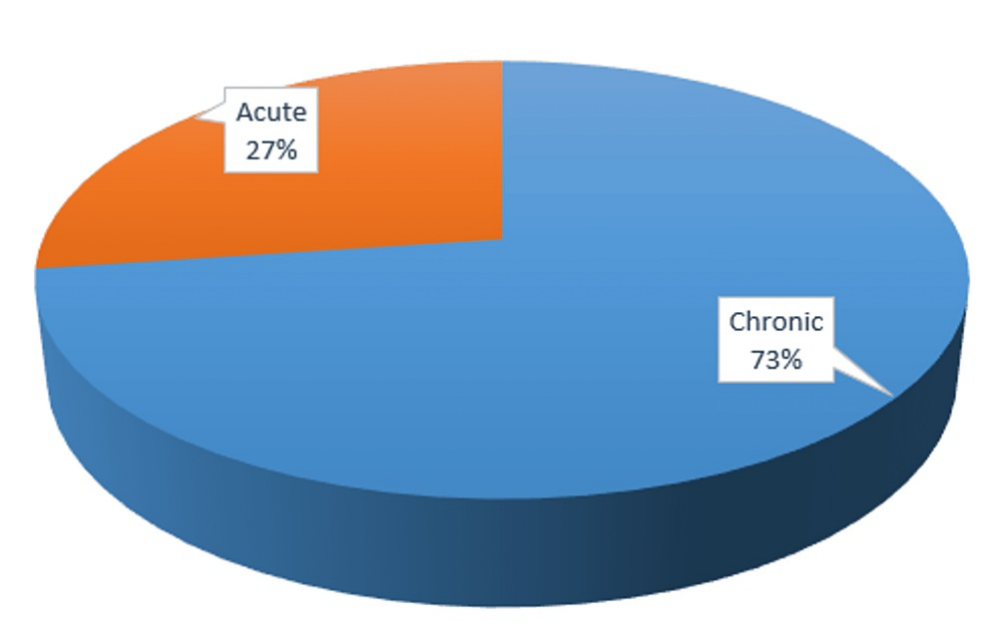
Observation of chronic and acute cholecystitis

**Figure 4:**
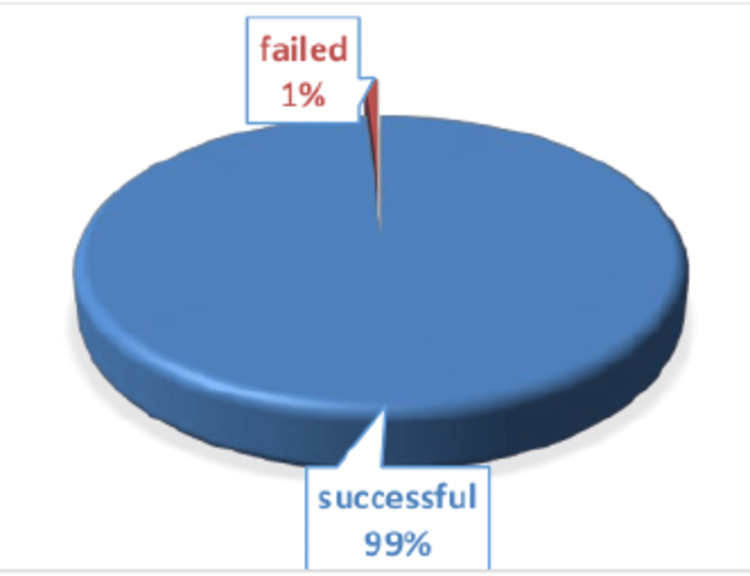
Success rate of the technique

All surgeries were performed during the first hospital admission of the patients. Successful extraction of the gallbladder was achieved in 540 (99%) cases. The remaining five (1%) cases had to be converted to open cholecystectomy due to frozen callots, difficult gallbladder anatomy, and extensive scaring. The biopsy specimen shown gall bladder carcinoma in those 1% cases (Figure 4).

There is no VBI or BDI noted. No mortality was recorded during the study period.

## Discussion

As lap chole's learning curve smoothed out, the incidence of VBI stayed under control and no such leak was seen throughout the entire trial period [8,9]. However, according to recent data, mortality and morbidity have not altered considerably, but BDI has been increasing for a number of years (0.32 to 0.52) [10,11]. Misidentification increases the incidence of VBI; the common bile duct is frequently mistaken for the cystic duct and, less frequently, aberrant hepatic ducts are mistaken for cystic ducts [12]. Although CVS can prevent VBI caused due to misidentification in complicated lap chole, it is not always practical because, until the cystic structure is removed, it is challenging to separate the gallbladder from the liver bed during CVS [13,14]. As a result, in difficult lap chole cases, where CVS is not attained, surgeons should think about an alternative treatment like open cholecystectomy [15]. In such cases, we suggest that rather than individualizing the procedure, CVS might be simply performed by observing the Triple One method. The surgeon will avoid misidentifying the cystic structures in order to get CVS by dissecting in accordance with the Triple One technique.

On the other hand, gallbladder wall perforations during dissection may indicate scarring of the gallbladder wall leading to VBI [16]. When severe scarring within the hepatocystic triangle prevented the dissection and release of various structures to achieve CVS, an open cholecystectomy is performed. In our study, in accordance with the decision criteria and the surgeon's discretion during gallbladder dissection, open cholecystectomy was carried out in about 1% of the challenging lap chole cases.

The Triple One strategy has a very small number of restrictions compared to other methods. This method is almost always appropriate even when the edge of the gallbladder is difficult to identify physically because of inflammatory adhesions with surrounding structures. In this study, our operating policy was to only perform open surgery where there was a very strong suspicion of malignancy and tough adhesion. 

Our technique is different from conventional technique in the following ways: (i) In this technique, the direction of the dissection is towards the gallbladder rather than going down towards CBD and vital structures; (ii) Lymph node of Lund is the starting point of dissection in our technique while in conventional method there is no fixed point of dissection to start with. However, R4U (Rouviere's sulcus→Segment 4→Umbilical fissure) line is the lower limit of dissection, which is observed in both; (iii) All the dissections can be done via hook cautery, which is easily available all over the world; (iv) Artery is medialised and ligated away from its origin, i.e. near the gallbladder wall, which is ligated near the origin in conventional technique.

The fact that none of our patients had any significant or small leaks during or after surgery proved the Triple One technique's efficiency in preventing BDI and VBI as well as the morbidity and mortality associated with them. Furthermore, there are no disadvantages to this technique as it is a modification and standardization of previously performed methods.

Our study was limited in that none of the operational notes addressed the Triple One approach or the timeout, even though photos were taken after the CVS. Another limitation was that our patients were aged 30-70 years. Also, patients with prior abdominal procedures were not included in this study. In future studies, this technique can be studied on a wider group of patients.

## Conclusions

During lap chole, it is thought that misidentification most frequently leads to BDI and VBI. One can achieve a critical view of safety by using the Triple One technique, which lowers the possibility of incidents like that. By incorporating CVS using the Triple One technique into our policies and curriculum, we may encourage safe cholecystectomy practices and prevent BDI. Long-term patient outcomes, comparison with traditional approaches, training and skills development programs, and adaptability to complex cases are some potential future implications and areas for further study that can showcase the wider relevance of this technique in the field of lap chole.

## References

[1] Brunt LM, Deziel DJ, Telem DA (2020). Safe cholecystectomy multi-society practice guideline and state of the art consensus conference on prevention of bile duct injury during cholecystectomy. Ann Surg.

[2] Kohn JF, Trenk A, Kuchta K (2018). Characterization of common bile duct injury after laparoscopic cholecystectomy in a high-volume hospital system. Surg Endosc.

[3] van de Graaf FW, Zaïmi I, Stassen LP, Lange JF (2018). Safe laparoscopic cholecystectomy: a systematic review of bile duct injury prevention. Int J Surg.

[4] Cengiz Y, Lund M, Jänes A, Lundell L, Sandblom G, Israelsson L (2019). Fundus first as the standard technique for laparoscopic cholecystectomy. Sci Rep.

[5] Sebastian M, Sroczyński M, Rudnicki J (2019). Using laparoscopic ultrasound to delineate dangerous anatomy during difficult laparoscopic cholecystectomies. Adv Clin Exp Med.

[6] Wakabayashi G, Iwashita Y, Hibi T (2018). Tokyo Guidelines 2018: surgical management of acute cholecystitis: safe steps in laparoscopic cholecystectomy for acute cholecystitis (with videos). J Hepatobiliary Pancreat Sci.

[7] Gené Škrabec C, Pardo Aranda F, Espín F, Cremades M, Navinés J, Zárate A, Cugat E (2020). Fluorescent cholangiography with direct injection of indocyanine green (ICG) into the gallbladder: a safety method to outline biliary anatomy. Langenbecks Arch Surg.

[8] Komaei I, Navarra G, Currò G (2017). Three-dimensional versus two-dimensional laparoscopic cholecystectomy: a systematic review. J Laparoendosc Adv Surg Tech A.

[9] Schwab KE, Curtis NJ, Whyte MB, Smith RV, Rockall TA, Ballard K, Jourdan IC (2020). 3D laparoscopy does not reduce operative duration or errors in day-case laparoscopic cholecystectomy: a randomised controlled trial. Surg Endosc.

[10] Kaya B, Fersahoglu MM, Kilic F, Onur E, Memisoglu K (2017). Importance of critical view of safety in laparoscopic cholecystectomy: a survey of 120 serial patients, with no incidence of complications. Ann Hepatobiliary Pancreat Surg.

[11] Cai XJ, Ying HN, Yu H (2015). Blunt dissection: a solution to prevent bile duct injury in laparoscopic cholecystectomy. Chin Med J (Engl).

[12] Nassar AH, Nassar MK, Gil IC, Ng HJ, Yehia AM (2021). One-session laparoscopic management of Mirizzi syndrome: feasible and safe in specialist units. Surg Endosc.

[13] Gupta V, Jain G (2019). Safe laparoscopic cholecystectomy: adoption of universal culture of safety in cholecystectomy. World J Gastrointest Surg.

[14] Vettoretto N, Saronni C, Harbi A, Balestra L, Taglietti L, Giovanetti M (2011). Critical view of safety during laparoscopic cholecystectomy. JSLS.

[15] Pucher PH, Brunt LM, Davies N (2018). Outcome trends and safety measures after 30 years of laparoscopic cholecystectomy: a systematic review and pooled data analysis. Surg Endosc.

[16] Bleszynski MS, DeGirolamo KM, Meneghetti AT, Chiu CJ, Panton ON (2020). Fluorescent cholangiography in laparoscopic cholecystectomy: an updated Canadian experience. Surg Innov.

